# 863. Pre-Exposure Prophylaxis (PrEP) Prescriptions among Individuals at High Risk for HIV in the United States, 2012-2018

**DOI:** 10.1093/ofid/ofab466.1058

**Published:** 2021-12-04

**Authors:** Mo Zhou, Yan Song, Emily Gao, Yohance Whiteside, Emma Billmyer, James Signorovitch

**Affiliations:** 1 Analysis Group, Inc., Boston, MA, USA; 2 Merck & Co., Inc., Philadelphia, Pennsylvania

## Abstract

**Background:**

To describe trends in emtricitabine and tenofovir disoproxil fumarate (FTC/TDF) PrEP uptake in 2012-2018 and characterize high risk individuals who use PrEP.

**Methods:**

The study identified individuals aged ≥ 15 years old with claims suggesting high risk for HIV infection in the IBM MarketScan® Commercial Claims and Multi-state Medicaid Databases. High risk was defined using ICD codes indicating high risk sexual behavior or rectal/repeated bacterial sexually transmitted infection (STI). The index date was defined as the earliest of the first high risk sexual behavior diagnosis, the first rectal bacterial STI diagnosis, or the second non-rectal bacterial STI diagnosis within 12 months. Individuals were considered PrEP users if they had at least one FTC/TDF PrEP prescription within 12 months of index date. Individuals with evidence of HIV prior to or within 30 days after PrEP initiation/index date were excluded. Comorbidities were assessed using a modified Charlson Comorbidity Index that excluded HIV/AIDS.

**Results:**

FTC/TDF PrEP uptake increased from 0.1% to 7.3% among commercially insured individuals between 2012-2018, and from 0.01% to 0.5% among Medicaid insured individuals between 2012-2017. Individuals ≥ 35 years old had the largest increase in PrEP uptake (0.1% to 13.0%), while those 16-25 years old had the smallest increase (0.03% to 2.3%). The largest proportion of PrEP users across all years were aged 25-34 while the largest proportion of non-PrEP users were aged 18-24. Compared to PrEP users, a larger proportion of PrEP non-users were female (62.9% vs. 1.4%, *p* < 0.05) and blacks/African American (49.1% vs. 40.3%, *p* < 0.05). A larger proportion of PrEP users had a risk status of homosexual (46.6% vs. 1.5%, *p* < 0.05) or bisexual (3.9% vs. 0.8%, *p* < 0.05) behavior than non-PrEP users. PrEP users also had more comorbidities than non-users among individuals with Medicaid and were less likely to have fee-for-service insurance plans overall (*p* < 0.05).

Table 1. Characteristics of people at high-risk for HIV who do vs. do not use FTC/TDF PrEP measured during the follow-up period

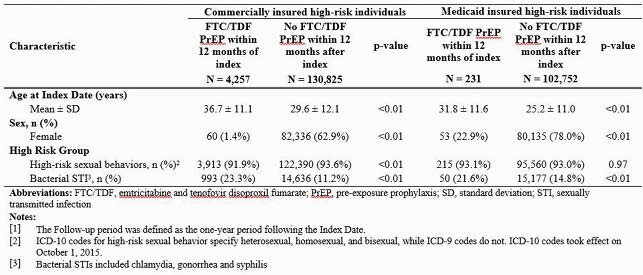

**Conclusion:**

Despite an increase in FTC/TDF PrEP initiations, uptake was low, especially among young adults, women, heterosexuals and blacks/African Americans. Low initiation rates in these groups may illustrate that FTC/TDF PrEP is not meeting the needs of all high-risk individuals.

**Disclosures:**

**Mo Zhou, PhD**, **Merck & Co., Inc.** (Consultant) **Yan Song, PhD**, **Merck & Co., Inc.** (Consultant) **Emily Gao, MS, MPH**, **Merck & Co., Inc.** (Consultant) **Yohance Whiteside, PhD, MSPH**, **Merck & Co., Inc.** (Employee) **Emma Billmyer, BA**, **Merck & Co., Inc.** (Consultant) **James Signorovitch, PhD**, **Merck & Co., Inc.** (Consultant)

